# Performance evaluation of early growth isolates for automated and manual broth microdilution antimicrobial-susceptibility testing

**DOI:** 10.1128/jcm.00236-25

**Published:** 2025-06-30

**Authors:** Lucas J. Osborn, Lindsay Osborn, Irvin Ibarra-Flores, Marisol Garcia, Kaitlyn Perez, Ali Farhadiayoubloo, Melissa Mitrou, Cristina Costales, Jennifer Dien Bard

**Affiliations:** 1 Department of Pathology and Laboratory Medicine, Children's Hospital Los Angeles 635205, Los Angeles, California, USA; 2 Keck School of Medicine, University of Southern California 12223, Los Angeles, California, USA; Johns Hopkins University, Baltimore, Maryland, USA

**Keywords:** antimicrobial-susceptibility testing, broth microdilution, rapid susceptibility, turnaround time, antibiotics

## Abstract

Prolonged turnaround times (TAT) represent a major limitation to current automated susceptibility testing systems and manual susceptibility-testing methods such as broth microdilution. As a result, targeted therapy for patients may be delayed, portending suboptimal clinical outcomes. One contributing factor is the 18–24 h of incubation prior to antimicrobial-susceptibility testing (AST) recommended by the Clinical Laboratory Standards Institute (CLSI) and some automated AST manufacturers. This study evaluates the performance of AST by manual broth microdilution (Thermo Fisher Sensititre) and an automated AST system (BD Phoenix) on isolates incubated for 6 h (early growth AST, egAST) compared with 18–24 h (standard growth AST, sgAST). An initial proof-of-concept study conducted on gram-negative (*n* = 5) and gram-positive (*n* = 2) quality control strains incubated for 6 or 24 h prior to Sensititre and Phoenix demonstrated 100% essential agreement. Subsequently, we evaluated the performance of egAST on gram-positive (*n* = 49, Phoenix; *n* = 46 Sensititre) and gram-negative (*n* = 81 Phoenix; *n* = 61 Sensititre) patient-derived isolates with diverse resistance profiles compared with sgAST. In total, 1,666 organism–drug combinations were tested by Sensititre (560 gram-positive and 1,106 gram-negative) and 1,927 by Phoenix (409 gram-positive and 1,518 gram-negative). For *Enterobacterales*, egAST using Phoenix revealed 1.3% minor errors (MiE), 0.17% major errors (ME), and 1.1% very major errors (VME) compared with sgAST. Phoenix egAST performance for *Pseudomonas aeruginosa* revealed 0.18% MiE and no ME or VME. Similarly, egAST of *Enterobacterales* by Sensititre revealed 1.5% MiE and no ME or VME, whereas 2.8% MiE, 6.6% ME, and no VME were observed for *P. aeruginosa*. For *Staphylococcus* spp. and *Enterococcus* spp., there were no MiE, ME, or VME on the Phoenix system, whereas early growth Sensititre showed 3.14% MiE, 0.3% ME, and 2.99% VME for *Staphylococcus* spp. and 6.8% MiE, 0.09% ME, and no VME for *Enterococcus* spp. Taken together, these data suggest that egAST represents a viable strategy to reduce the prolonged incubation period currently recommended by CLSI and select automated AST manufacturer guidelines without incurring any additional costs while simultaneously maintaining high concordance with reference standard methods.

**IMPORTANCE:**

Traditional antimicrobial-susceptibility testing (AST) methods typically span several days from the time of organism isolation. The majority of this time is spent waiting for a cultured isolate to incubate up to 1 day prior to AST. There exists an unmet need to provide more rapid AST as various rapid methods have been shown to reduce exposure to broad-spectrum antibiotics that select for antimicrobial resistance, shorten hospital stays, and improve clinical outcomes. Simultaneously, there is a need to ensure that rapid AST approaches are readily implemented in the clinical microbiology laboratory, with little to no added financial burden. This study demonstrates a cost-considerate and practical approach to reduce AST turnaround times by up to 18 h through the use of early growth isolates in combination with two commercial AST systems. The findings from this study complement those of previous reports describing the largely acceptable performance of disk diffusion using early growth isolates.

## INTRODUCTION

Antimicrobial-susceptibility testing (AST) is an important component of clinical microbiology and the treatment and management of bacterial infectious diseases (1–3). Antimicrobial susceptibilities can be measured using a variety of methodologies, including manual disk diffusion (DD) and broth microdilution methods (BMDs) as well as commercially available automated systems (4). Manual BMD (Sensititre) involves preparing a standardized suspension of a bacterial isolate, which is subsequently inoculated into a microtiter plate containing wells dosed with antimicrobial agents at various dilutions (5). The plate is visually inspected for growth to determine the minimum inhibitory concentration (MIC) (5). With the increasing use of automation in the laboratory, automated AST systems have proven to be beneficial to the microbiology workflow. Our laboratory employs automated AST with the BD Phoenix M50 (Becton Dickinson, Sparks, MD, USA), which offers both gram-positive and gram-negative panels. The Phoenix performs susceptibility testing through the determination of bacterial growth in the presence of various concentrations of antimicrobial agents by measuring redox reactions and turbidity (5, 6).

Currently, Sensititre and Phoenix methods require prolonged incubation of an isolated microorganism before susceptibility testing may be performed per Clinical and Laboratory Standards Institute (CLSI) guidelines (7). These guidelines, as well as some manufacturer instructions for use, recommend using pure bacterial colonies incubated for 18–24 h on non-selective agar (5–8). From organism isolation to final AST results, the turnaround time (TAT) for this process in its entirety may approach 48 h. Reducing the time to AST results allows for prompt optimization of patient management (9, 10). Clinicians follow empiric treatment protocols while waiting for susceptibility results, which include broad-spectrum coverage that may contribute to antimicrobial resistance when used over long periods of time (1). A shorter TAT for antimicrobial susceptibilities empowers clinicians to prescribe appropriate, more targeted therapy for patients sooner (9–12). This is especially beneficial for organisms that exhibit resistance patterns not covered by empiric treatment (2).

Several previous studies have explored the performance of early growth AST (egAST) using DD methods (4, 13, 14). These studies generally demonstrate strong agreement between early growth and reference standard methodologies (sgAST) (4, 13, 14). The results from these studies provide evidence to support updated susceptibility standards and guidelines for egAST DD in the future. However, the performance of BMD and automated AST methods using isolates < 18 h old remains unclear. Here, we investigate the performance of egAST compared with the reference standard using Phoenix and Sensititre methods. Our work seeks to fill the current gap in the literature for egAST using these methods and may ultimately support adoption within the clinical laboratory.

## MATERIALS AND METHODS

### Bacterial isolates

Gram-positive (*n* = 2) and gram-negative (*n* = 5) quality control (QC) strains and gram-positive (*n* = 49, Phoenix; *n* = 46 Sensititre) and gram-negative (*n* = 81 Phoenix; *n* = 61 Sensititre) patient-derived isolates with diverse resistance profiles were selected for use as representative organisms commonly encountered in the clinical laboratory. For the initial Phase I feasibility study, gram-positive QC isolates included *Staphylococcus aureus* ATCC 29213 and *Enterococcus faecalis* ATCC 29212. Gram-negative QC isolates included *Escherichia coli* ATCC 25922 and 35218, *Pseudomonas aeruginosa* ATCC 27853, *Proteus mirabilis* ATCC 12453, and *Klebsiella pneumoniae* ATCC 700603. Phase II clinical isolates included various members of the order *Enterobacterales*, *P. aeruginosa, S. aureus, E. faecalis, E. faecium,* and *Staphylococcus* spp. other than *S. aureus* (SOSA). All organisms were inoculated onto trypticase soy agar with sheep blood (Becton Dickinson) using a four-quadrant streaking method and incubated for 6 h or 24 h at 35°C prior to setting up both Phoenix and Sensititre with one plate for each time point.

### Phoenix testing

The BD Phoenix M50 PMIC-108 and NMIC-311 panels were used for gram-positive and gram-negative organisms, respectively. A 4.5 mL suspension of all clinical and QC organisms was prepared and adjusted to a concentration of 0.5 McFarland on the BD Phoenix AP instrument. PMIC-108 and NMIC-311 panels were prepared and incubated per the manufacturer’s protocol (6). Incubation and reagent panel interpretation occurred directly within the instrument. A purity plate for each isolate was planted to examine for potential contamination.

### Sensititre testing

In conjunction with Phoenix testing, all isolates were also tested using the Sensititre System (Thermo Fisher Scientific, Walham, MA, USA). Suspensions of the clinical and QC isolates were inoculated according to the manufacturer’s protocol. Briefly, pure isolates were selected to make a 0.5 McFarland suspension in Sensititre demineralized water. The suspensions were read on the Sensititre nephelometer to ensure the correct concentration was achieved. Ten microliters (1 µL if *Proteus* spp.) of the McFarland suspension were transferred into an 11 mL tube of cation-adjusted Mueller Hinton broth with TES buffer (Thermo Fisher Scientific). The cation-adjusted tube with bacterial suspension was inoculated using the Thermo Scientific Sensititre AIM Automated Inoculation Delivery System.

Either GNX2F or GPALL3F microtiter plates were used for gram-negative or gram-positive organisms, respectively. Once AST was set up, all organisms were incubated aerobically at 35°C for the appropriate amount of time as described in the manufacturer’s protocol. A purity plate was included to examine for potential contamination.

### Data analysis

The AST results of 6 h early growth isolates were compared with those of the reference 24 h incubation method for both Sensititre and Phoenix using the most current CLSI breakpoints (15). Any very major errors from gram-negative isolates were repeated in triplicate. The performance of the early growth isolates for both AST methods was analyzed for the number and types of errors, as well as essential agreement (EA), when the MIC matches between the test and reference methods within one doubling dilution, and categorical agreement (CA), when the MIC interpretation of susceptible (S), intermediate (I), or resistant (R) is equivalent between methods for a particular antimicrobial (16). Minor errors (MiE) were defined as when an organism’s susceptibility interpretation is intermediate (I) but reported as S or R by the test method. Conversely, a minor error also occurs when an organism is S or R to a particular antimicrobial agent but is reported as I. Major errors (ME) occur when an organism is reported as having false resistance, and very major errors (VME) occur when an organism is classified as being falsely susceptible to a drug in which it demonstrates resistance by the reference standard.

### Calculations and statistics

EA, CA, MiE, ME, and VME were calculated according to the standards defined by the Food and Drug Administration’s guidance document for antimicrobial susceptibility testing (17). For data visualization purposes, *Enterobacterales* with cefepime and piperacillin-tazobactam MIC interpretations of susceptible dose-dependent (SDD) were treated as I. The confidence intervals listed in Tables 1 to 4 were calculated using the Microsoft Excel’s “=CONFIDENCE” formula. Only on-scale EA results were included (i.e., evaluable results rather than overall EA).

**TABLE 1:** Performance of early growth automated antimicrobial susceptibility testing of gram-negative rods with the Phoenix M50^*a*^^,^^*b*^^,^^*d*^

*Enterobacterales*									
	Number of isolates tested				Agreement (%)		No. of errors, n/N (%)		
Antibiotic	Total	S	I	R	EA*	CA	MiE	ME	VME
Amoxicillin-Clavulanate	62	47	13	2	100	100			
Ampicillin	54	18	0	36	^*c*^–	100			
Ampicillin-sulbactam	58	22	16	20	100	100			
Cefazolin	62	34	8	20	97.1	91.9	5/62 (8.1)		
Cefepime**	71	66	2	3	80	98.6	1/71 (1.4)		
Cefoxitin	62	60	1	1	100	100			
Ceftazidime**	71	66	0	5	50	97.1		1/66 (1.5)	1/5 (20.0)
Ceftazidime-avibactam	71	71	0	0	100	100			
Ceftolozane-tazobactam**	71	69	0	2	100	100			
Ceftriaxone	71	63	0	8	–	100			
Cefuroxime**	62	54	1	7	100	100			
Ciprofloxacin	71	50	8	13	100	92.9	5/71 (7.0)		
Ertapenem**	71	70	1	0	100	98.6	1/71 (1.4)		
Gentamicin**	71	59	1	11	100	98.6	1/71 (1.4)		
Imipenem	58	58	0	0	100	100			
Levofloxacin	71	59	7	5	100	98.6	1/71 (1.4)		
Meropenem	67	67	0	0	–	100			
Meropenem-vaborbactam	71	71	0	0	–	100			
Piperacillin-tazobactam	71	62	3	6	93.3	97.2	1/71 (1.4)	1/62 (1.6)	
Tobramycin	71	58	0	13	100	95.8	3/71 (4.2)		
Trimethoprim-sulfamethoxazole**	71	38	0	33	0	98.6			1/33 (3.0)
Total	1408	1162	61	185	97.5	98.4	18/1408 (1.3)	2/1162 (0.17)	2/185 (1.1)
Sample size					203	1408			
Standard deviation					25.5	2.3			
Alpha					0.05	0.05			
Confidence					3.51	0.12			
5% confidence					94	98.3			
95% confidence					101	98.6			

***Pseudomonas aeruginosa***									
	Number of isolates tested				Agreement (%)		No. of errors, n/N (%)		
Antibiotic	Total	S	I	R	EA*	CA	MiE	ME	VME
Amikacin	10	9	0	1	100	100			
Aztreonam	10	7	1	2	90	90	1/10 (10)		
Cefepime	10	6	1	3	100	100			
Ceftazidime	10	7	1	2	100	90	1/10 (10)		
Ceftazidime-avibactam	10	10	0	0	100	100			
Ceftolozane-tazobactam	10	8	0	2	100	100			
Ciprofloxacin	10	7	0	3	100	100			
Imipenem	10	6	2	2	100	100			
Levofloxacin	10	7	0	3	100	100			
Meropenem	10	9	0	1	100	100			
Piperacillin-tazobactam	10	8	1	1	100	100			
Total	110	84	6	20	98.7	98.2	2/110 (0.18)	0/84 (0.0)	0/20 (0.0)
Sample size					83	110			
Standard deviation					2.87	3.86			
Alpha					0.05	0.05			
Confidence					0.62	0.72			
5% confidence					98.1	97.5			
95% confidence					99.3	98.9			

^*a*^ *Only isolates with on-scale MIC included.

^*b*^ **≤ 5 isolates with on-scale MIC.

^*c*^ Unable to calculate EA as there were no on-scale MIC.

^*d*^ Empty cells indicates there were no errors thus no subsequent calculations of summary statistics.

**TABLE 2:** Performance of early growth manual antimicrobial susceptibility testing of gram-negative rods by Sensititre broth microdilution^*a*^*^,^*^*b*^*^,^*^*e*^

Enterobacterales									
	Number of isolates tested				Agreement (%)		No. of errors, *n/N* (%)		
Antibiotic	Total	S	I	R	EA*	CA	MiE	ME	VME
Amikacin**	51	49	1	1	100	98.0	1/51 (1.9)		
Aztreonam**	51	47	0	4	100	100			
Cefepime**	51	49	0	2	100	96.1	2/51 (3.9)		
Cefotaxime	51	46	0	5	^*c*^–	100			
Ceftazidime**	51	47	0	4	100	98.0	1/51 (1.9)		
Ciprofloxacin**	51	43	2	6	100	100			
Colistin**	47	0	47	0	–	100			
Doripenem**	51	51	0	0	100	100			
Doxycycline	51	34	2	15	100	98.0	1/51 (1.9)		
Ertapenem	51	51	0	0	–	100			
Gentamicin**	51	47	0	4	100	100			
Imipenem**	51	50	1	0	100	100			
Meropenem	51	51	0	0	–	100			
Minocycline	51	38	5	8	100	94.1	3/51 (5.9)		
Piperacillin-tazobactam**	51	45	1	5	100	100			
Polymixin B	47	0	47	0	100	100			
Ticarcillin-Clavulanic acid	51	39	6	6	100	88.2	6/51 (11.8)		
Tobramycin**	51	47	1	3	100	100			
Trimethoprim-sulfamethoxazole**	51	36	0	15	100	100			
Total	961	770	113	78	100	98.6	14/961 (1.5)	0/770 (0.0)	0/78 (0.0)
Sample size*					60	961			
Standard deviation					0.00	2.91			
Alpha					0.05	0.05			
Confidence					^*d*^NC	0.18			
5% confidence					NC	98.4			
95% confidence					NC	98.8			

*Pseudomonas aeruginosa*									
	Number of isolates tested				Agreement (%)		No. of errors, *n/N* (%)		
Antibiotic	Total	S	I	R	EA*	CA	MiE	ME	VME
Amikacin**	10	9	0	1	100	100			
Aztreonam**	10	8	0	2	100	100			
Cefepime**	9	6	1	2	100	100			
Ceftazidime**	10	8	0	2	40.0	100			
Ciprofloxacin**	10	7	1	2	100	100			
Colistin	10	0	10	0	100	100			
Doripenem**	10	10	0	0	100	70.0		3/10 (30.0)	
Imipenem**	9	4	2	3	33.3	55.5	3/9 (33.3)	1/4 (25.0)	
Levofloxacin	7	7	0	0	–	100			
Meropenem**	10	9	1	0	33.3	80.0	1/10 (10.0)	1/9 (11.1)	
Minocycline	10	0	0	10	–	100			
Piperacillin-tazobactam**	10	9	0	1	100	100			
Polymixin B	10	0	10	0	100	100			
Ticarcillin-clavulanic acid**	10	6	2	2	33.3	90.0		1/6 (16.7)	
Tobramycin	10	8	0	2	–	100			
Total	145	91	27	27	90.2	93.1	4/145 (2.8)	6/91 (6.6)	0/27 (0.0)
Sample size					51	145			
Standard deviation					30.70	13.30			
Alpha					0.05	0.05			
Confidence					8.43	2.16			
5% confidence					81.8	90.9			
95% confidence					98.6	95.3			

^*a*^ *Only isolates with on-scale MIC included.

^*b*^ **≤ 5 isolates with on-scale MIC.

^*c*^ Unable to calculate EA as there were no on-scale MIC.

^*d*^ Not calculated due to 100% agreement.

^*e*^ Empty cells indicates there were no errors thus no subsequent calculations of summary statistics.

**TABLE 3:** Performance of early growth automated antimicrobial susceptibility testing of gram-positive cocci with the Phoenix M50^*a*^*^,^*^*b*^*^,^*^*e*^

*Staphylococcus* spp.									
	Number of isolates tested				Agreement (%)		No. of errors, *n/N* (%)		
Antibiotic	Total	S	I	R	EA*	CA	MiE	ME	VME
Cefoxitin**	24	10	0	14	100	100			
Clindamycin	33	25	0	8	^*c*^–	100			
Daptomycin	33	33	0	0	–	100			
Erythromycin	33	12	0	21	–	100			
Linezolid	33	33	0	0	100	100			
Minocycline**	33	33	0	0	100	100			
Oxacillin**	33	17	0	16	100	100			
Rifampin	33	33	0	0	–	100			
Tetracycline**	33	30	1	2	100	100			
Trimethoprim-sulfamethoxazole	24	22	0	2	–	100			
Vancomycin	33	33	0	0	100	100			
Total	345	281	1	63	100	100	0/345 (0.0)	0/281 (0.0)	0/63 (0.0)
Sample size					44	345			
Standard deviation					0.00	0.00			
Alpha					0.05	0.05			
Confidence					^*d*^NC	NC			
5% confidence					NC	NC			
95% confidence					NC	NC			

*Enterococcus* spp.									
	Number of isolates tested				Agreement (%)		No. of errors, *n/N* (%)		
Antibiotic	Total	S	I	R	EA*	CA	MiE	ME	VME
Ampicillin	16	15	0	1	100	100			
Penicillin G	16	15	0	1	100	100			
Linezolid**	16	16	0	0	100	100			
Vancomycin	16	15	0	1	100	100			
Total	64	61	0	3	100	100	0/64 (0.0)	0/61 (0.0)	0/3 (0.0)
Sample size					40	64			
Standard deviation					0.00	0.00			
Alpha					0.05	0.05			
Confidence					NC	NC			
5% confidence					NC	NC			
95% confidence					NC	NC			

^*a*^ *Only isolates with on-scale MIC included.

^*b*^ **≤ 5 isolates with on-scale MIC.

^*c*^ Unable to calculate EA as there were no on-scale MIC.

^*d*^ Not calculated due to 100% agreement.

^*e*^ Empty cells indicates there were no errors thus no subsequent calculations of summary statistics.

**TABLE 4:** Performance of early growth manual antimicrobial susceptibility testing of Gram positive cocci by Sensititre broth microdilution^*a*^^,*b*,*d*^

*Staphylococcus* spp.									
	Number of isolates tested				Agreement (%)		No. of errors, *n/N* (%)		
Antibiotic	Total	S	I	R	EA*	CA	MiE	ME	VME
Penicillin G**	8	0	0	8	80.0	100			
Ceftaroline	19	10	0	9	100	100			
Ciprofloxacin**	29	19	0	10	100	96.6	1/29 (3.45)		
Clindamycin**	29	21	0	8	0	89.7		1/21 (4.76)	2/8 (25.0)
Chloramphenicol	29	20	7	2	96.2	75.9	7/29 (24.1)		
Daptomycin	29	29	0	0	^*c*^–	100			
Erythromycin	29	9	3	17	–	89.7	3/29 (10.3)		
Gentamicin**	29	28	1	0	100	100			
Levofloxacin**	29	20	0	9	100	100			
Linezolid	29	29	0	0	100	100			
Oxacillin**	20	18	0	2	100	100			
Rifampin	29	27	2	0	–	93.1	2/29 (6.89)		
Telavancin	19	19	0	0	100	100			
Tetracycline**	29	26	1	2	100	100			
Trimethoprim-sulfamethoxazole	29	29	0	0	–	100			
Vancomycin	29	29	0	0	100	100			
Total	414	333	14	67	97.0	96.1	13/414 (3.14)	1/333 (0.30)	2/67 (2.99)
Sample size					132	414			
Standard deviation					27.59	6.44			
Alpha					0.05	0.05			
Confidence					4.71	0.62			
5% confidence					92.3	95.5			
95% confidence					101.7	96.8			

*Enterococcus* spp.									
	Number of isolates tested				Agreement (%)		No. of errors, *n/N* (%)		
Antibiotic	Total	S	I	R	EA*	CA	MiE	ME	VME
Ampicillin	17	16	0	1	81.8	100			
Penicillin G	17	15	0	2	100	100			
Chloramphenicol	17	13	2	2	81.8	72.7	3/17 (17.6)	1/13 (7.7)	
Daptomycin**	17	17	0	0	100	100			
Erythromycin**	17	2	10	5	81.8	72.7	6/17 (35.3)		
Linezolid	17	16	1	0	100	90.9	1/17 (5.9)		
Rifampin**	17	6	2	9	100	100			
Telavancin	10	10	0	0	90.0	100			
Vancomycin	17	16	0	1	90.9	100			
Total	146	111	15	20	88.4	92.5	10/146 (6.8)	1/111 (0.09)	0/20 (0.0)
Sample size					95	146			
Standard deviation					7.98	11.17			
Alpha					0.05	0.05			
Confidence					1.60	1.81			
5% confidence					86.8	90.7			
95% confidence					90.0	94.3			

^*a*^ *Only isolates with on-scale MIC included.

^*b*^ **≤ 5 isolates with on-scale MIC.

^*c*^ Unable to calculate EA as there were no on-scale MIC.

^*d*^ Empty cells indicates there were no errors thus no subsequent calculations of summary statistics.

## RESULTS

The results of the Phase I feasibility study using QC strains showed 100% EA between 6 h egAST and 24 h sgAST isolates (Tables S1 & S2). These data suggested that there was utility in conducting a larger-scale Phase II study using clinical isolates.

**Fig 1:**
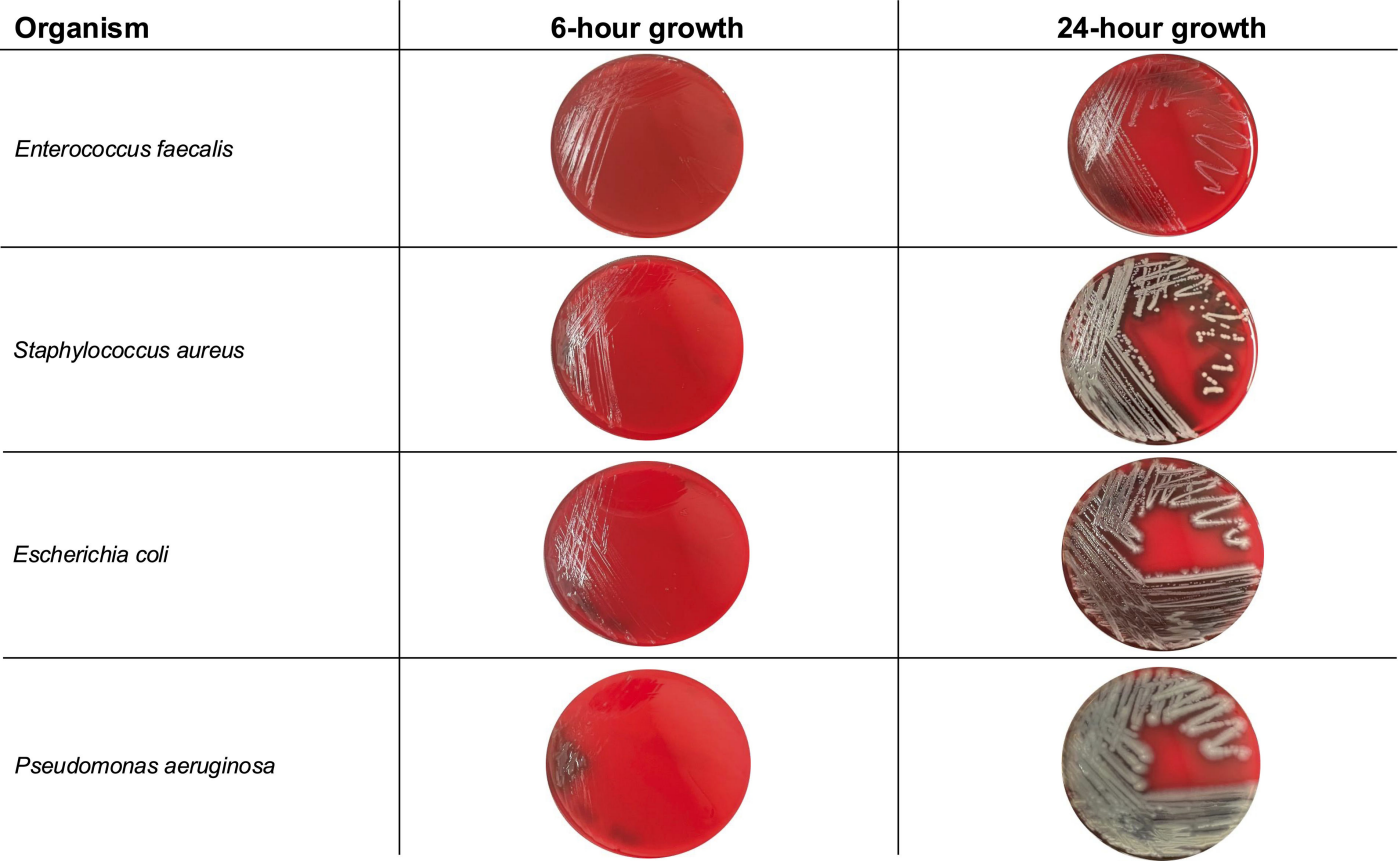
Growth of representative cultures on blood agar plates after 6 and 24 hours of incubation. Trypticase soy agar with sheep blood plates incubated at 37°C for either 6 or 24 h.

In total, 142 clinical isolates from a pediatric quaternary care facility were selected for egAST and egAST by Phoenix and Sensititre. Here, we observed sufficient growth to perform AST in 91.6% of isolates after just 6 h of incubation, resulting in 130 isolates with AST results (Fig. 1, Table S3 & S4). Organisms with repeatedly insufficient growth after 6 h included *Proteus* spp. and *Staphylococcus* spp. other than *S. aureus*, *P. aeruginosa*, and *E. faecium* listed in the descending order of sufficient growth rate.

### Early growth AST performance by Phoenix

In total, 1,408 *Enterobacterales* drug-organism combinations were tested using Phoenix on early growth; resulting in EA and CA of 97.5% (95% CI: 94.0—101.0) and 98.4% (95% CI: 98.3—98.6), respectively (Table 1). Compared with the reference method, early growth gram-negative isolates by Phoenix resulted in 1.3% MiE, 0.17% ME, and 1.1% VME. Out of 110 organism-drug combinations tested for *P. aeruginosa,* egAST isolates demonstrated EA and CA of 98.7 (95% CI: 98.1—99.3) and 98.2 (95% CI: 97.5—98.9), respectively, with 0.18% MiE and no ME or VME.

*Staphylococcus* spp. and *Enterococcus* spp. early growth isolates on Phoenix resulted in no MiE, ME, or VME with 100% EA and CA (Table 3) on 409 drug-organism combinations.

### Early growth AST performance by Sensititre

Out of 961 *Enterobacterales* organism-drug combinations tested on early growth gram-negative isolates by Sensititre, there were 1.5% MiE, no ME, and no VME when compared with the reference method (Table 2). These data correspond to EA and CA of 100% and 98.6% (95% CI: 98.4—98.8), respectively. Out of 145 organism-drug combinations, early growth *P. aeruginosa* egAST isolates demonstrated 2.8% MiE, 6.6% ME, and no VME with 90.2% EA (95% CI: 81.8—98.6) and 93.1% CA (95% CI: 90.9—95.3).

Susceptibility testing of 414 organism-drug combinations of early growth *Staphylococcus* spp. isolates by Sensititre revealed 3.14% MiE, 0.3% ME, and 2.99% VME (Table 4). These data correspond to 97.0% (95% CI: 92.3—101.7) and 96.1% (95% CI: 95.5—96.8) EA and CA, respectively. Moreover, 145 organism-drug combinations were tested for *Enterococcus* spp egAST, revealing 6.8% MiE, 0.09% ME, and no VME with 88.4% (95% CI: 86.8—90.0) and 92.5% (95% CI 90.7—94.3) EA and CA, respectively.

## DISCUSSION

The standard of performing AST on isolates incubated for 18–24 h is not a data-driven recommendation but rather a relic of the past where many clinical microbiology laboratories performed AST only during routine business hours. In an ever-evolving landscape of advancements in diagnostic capabilities, laboratory consolidation, and volume-driven reimbursement metrics, many clinical microbiology laboratories now offer full-service testing around the clock (18). Secondary to this paradigm shift, there has been a push for more rapid susceptibility testing through both phenotypic and genotypic characterization. Rapid AST methods have already demonstrated tangible improvements in patient care by a reduction in empiric antibiotic exposure, reduced length of hospital stay, and improved mortality (19–22). These improvements are due in part to a reduced time to optimal therapy and are largely dependent on clinical action driven by a dedicated antimicrobial stewardship program (19, 21, 23). However, current approaches to reducing time to AST reporting require large capital expenditures for new equipment and reagents that may be cost-prohibitive for some laboratories. Consequently, there is an unmet need for a rapid AST method that can be broadly implemented in any laboratory currently performing AST without additional equipment, personnel, or reagents.

One approach to address the need for more rapid, cost-efficient AST is performing testing using the same materials and methodologies currently implemented in the laboratory, but with “early growth” isolates. Several studies to date have examined the performance of DD testing by egAST using isolates incubated for as little as 6 h (4, 13, 14). Herein, we describe the first application of egAST for broth microdilution and automated susceptibility testing leveraging the Sensititre and Phoenix systems, respectively. In this study, egAST CA exceeded the minimum 90% threshold set by the FDA Class II Special Controls Guidance for AST systems for both gram-negative and gram-positive isolates, regardless of testing platform or organism group (17). With the exception of *Enterococcus* spp. tested by Sensititre, greater than 90% overall EA was observed for both gram-negative and gram-positive organisms, regardless of the test system. Despite excellent overall CA and EA, several isolated organism-drug combinations exceeded the CLSI/FDA-recommended error thresholds, including doripenem, imipenem, meropenem, and ticarcillin-clavulanic acid by Sensititre for *P. aeruginosa*; ticarcillin-clavulanic acid by Sensititre for *Enterobacterales;* ceftazidime for *Enterobacterales* by Phoenix; erythromycin and chloramphenicol for all gram-positive organisms by Sensititre, and clindamycin for *Staphylococcus* spp. by Sensititre. With the exception of these isolated errors, the overall rates of MiE, ME, and VME for both BMD and automated AST systems were below the minimum criteria recommended by the CLSI (16, 24). These data are largely concordant with findings reported by other laboratories performing DD egAST (4, 13, 14), suggesting the performance of egAST is not limited to a single testing methodology.

Notably, more than 90% of isolates tested had sufficient growth to set up BMD or automated AST after just 6 h of incubation. For isolates with insufficient growth at 6 h, an additional 4 h of incubation may be required to produce enough colonies to yield a 0.5 McFarland inoculum. This approach has been tested by Webber et al. using DD, and similar performances of 6 h egAST and 10 h egAST were reported (4).

One limitation of this study is the exclusion of levofloxacin for *Enterobacterales* from BMD performance analysis as a recent revision to the breakpoints led to an off-scale susceptible breakpoint on the Sensititre GNX2F AST plate (15). Similarly, recent changes to aminoglycoside breakpoints precluded analysis of tobramycin for *P. aeruginosa* or amikacin for *Enterobacterales* on the BD Phoenix M50 NMIC-311 system. These limitations underscore challenges within the clinical microbiology laboratory where the current regulatory landscape requires validation of new breakpoints at a frequency that often exceeds reagent updates by AST system manufacturers (25). One additional limitation of this study is the relatively low rate of antimicrobial resistance among our pediatric population when compared with the adult population (26). Consequently, limited conclusions can be drawn from the performance analysis of isolates with low resistance rates. Although this study aimed to test the performance of egAST on isolates representative of our patient population, future studies should include isolates with well-characterized resistance profiles.

The CLSI and European Committee on Antimicrobial Susceptibility Testing (EUCAST) have published guidelines for rapid AST directly from blood culture (15, 27, 28). This rapid approach utilizes DD directly from the positive blood culture, and incubation times are organism and antibiotic-specific. Notably, the breakpoints established using rapid direct from blood culture methods differ from those using the reference standard method, whereas egAST methods from isolated pure cultures utilize reference standard breakpoints. Clinical laboratories lean on organizations such as CLSI and EUCAST to continuously evaluate innovations in AST and update or create new guidance documents when data support a new methodology. The data reported herein and concomitant egAST DD studies provide a foundation for organizations such as the CLSI and the EUCAST to evaluate, validate, and publish guidelines for egAST using various methodologies (4, 13, 14).

In summary, as clinical microbiology laboratories face unprecedented staffing shortages and financial headwinds, rapid diagnostic innovations must be cost-efficient and require no additional laboratory personnel (29–31). Implementation of egAST within the clinical microbiology laboratory allows for organism isolation, subculture, and AST setup within a single shift. Although egAST implementation would require method validation by clinical microbiology laboratories as it deviates from the FDA-cleared testing system protocol, the cost and time would be magnitudes less than adopting a new rapid AST system. This study highlights the potential for egAST by BMD or automated susceptibility platforms to reduce AST TAT by as much as 18 h without any additional financial or personnel requirements and with equivalent performance to the historical standard incubation time.

## SUPPLEMENTAL MATERIAL

Supplemental tables jcm.00236-25-s0001.xlsx Tables S1 to S4.

ASM does not own the copyrights to Supplemental Material that may be linked to, or accessed through, an article. The authors have granted ASM a non-exclusive, world-wide license to publish the Supplemental Material files. Please contact the corresponding author directly for reuse.
